# Draft genome sequences of representative *Paenibacillus polymyxa*, *Bacillus cereus*, *Fictibacillus* sp., and *Brevibacillus agri* strains isolated from Amazonian dark earth

**DOI:** 10.1128/MRA.00574-23

**Published:** 2023-10-09

**Authors:** Thierry Alexandre Pellegrinetti, Izadora De Cássia Mesquita da Cunha, Miriam Gonçalves de Chaves, Anderson Santos de Freitas, Ana Vitória Reina da Silva, Siu Mui Tsai, Lucas William Mendes

**Affiliations:** 1 Cell and Molecular Biology Laboratory, Center for Nuclear Energy in Agriculture, University of Sao Paulo, Piracicaba, Brazil; 2 Luiz de Queiroz College of Agriculture (ESALQ), University of São Paulo, Piracicaba, Brazil; DOE Joint Genome Institute, Berkeley, California, USA

**Keywords:** plant-microbe interaction, rhizosphere-inhabiting microbes, *Fusarium*, terra preta

## Abstract

Here, we report 10 distinct bacterial genomes from Amazonian dark earths, including six identified as *Paenibacillus polymyxa*, while the remaining four were unique representatives of *Paenibacillus vini*, *Bacillus cereus*, *Brevibacillus agri*, and *Fictibacillus* sp., respectively. Each strain exhibited antagonistic activity against *Fusarium oxysporum*, underscoring their potential as sustainable agriculture resources.

## ANNOUNCEMENT

Amazonian dark earths (ADEs), a fertile soil type formed by the management practices of pre-Columbian indigenous near Amazonian rivers over two millennia ago (1), is known for promoting plant growth and nutrient retention. Its diverse microbial communities play key roles in nutrient cycling and soil fertility, offering the potential for agricultural and ecological advancements (2). Microbial isolation from ADE allows for a detailed study of the soil’s distinctive chemical and biological properties. This understanding paves the way for insights into the ecological roles of resident microorganisms and their potential biotechnological applications in areas such as sustainable agriculture, waste management, and biofuel production (3).

The strains presented here were previously isolated in 2016 from an experiment using ADE soils and two different cultivars of common bean, exhibiting contrasting levels of resistance to the soil-borne pathogen *Fusarium oxysporum*. The experiment was conducted at the Center for Nuclear Energy in Agriculture of the University of São Paulo (22° 42*'* 27.60*"* S, 47° 38*'* 41.17*"* W) (4). Plants were collected at the R1 development stage (early flowering), and the roots were shaken to remove loosely adhering soil. The firmly attached soil was collected with sterile brushes and considered to be the rhizosphere soil. For microbial isolation, 1 g of rhizosphere soil was mixed with 9 mL of saline solution (8.5 g L-1 NaCl). Serial dilutions (10^−1^ to 10^−6^) were performed and then transferred onto KING medium plates (5). After incubating at 25°C for 48 h, colonies were isolated using the streak plate method. Total DNA from isolates was extracted using the DNeasy Ultraclean Microbial Kit (Qiagen Mobio, Hilden, Germany) following the manufacturer’s instructions.

Whole-genome sequencing was performed using the Illumina HiSeq 2500 platform. Genomic DNA was used for paired-end libraries (2 × 150 bp) with NEBNext Fast DNA Fragmentation and Library Preparation Kit (New England Biolabs Inc., MA, USA). The number of library reads is described in Table 1. We visualized the quality of raw sequences obtained from Illumina HiSeq sequencing by using FastQC v0.12.1 (6). Given that all raw reads exhibited a quality score exceeding Q30, trimming was not performed. Then, the sequences underwent *de novo* assembly using SPAdes v3.15.5 (7). We employed QUAST v4.4 (8) to measure the assembled genomes’ quality metrics. Paired-end reads were aligned to the assemblies using the Burrows-Wheeler Aligner tool v0.7.17 (9). The average sequencing coverage was then calculated from the resulting BAM (Binary Alignment Map) file using SAMtools v1.15 (10). Taxonomic classification was performed with GTDB-tk v.2.3.2 (11). The genome annotation was performed by NCBI Prokaryotic Genome Annotation Process v6.5 (12). An additional functional gene annotation was carried out using RAST-tk 1.3.0 (13). Default parameters were used for all software. Genome assembly statistics and information about the strains are summarized in Table 1.

**TABLE 1:** Assembly properties of 10 strains isolated from Amazonian dark earth, including contig statistics, genome size, GC content, and predicted gene count

	*P. vini*	*P. polymyxa*	*P. polymyxa*	*Fictibacillus* sp*.*	*B. agri*	*P. polymyxa*	*B. cereus*	*P. polymyxa*	*P. polymyxa*	*P. polymyxa*
Isolate	CENA-BCM001	CENA-BCM002	CENA-BCM003	CENA-BCM004	CENA-BCM005	CENA-BCM006	CENA-BCM007	CENA-BCM008	CENA-BCM009	CENA-BCM010
Library size (million of reads)	20.9	23.2	25.7	24	26.8	21.6	19.6	24.4	26.7	22.3
Number of contigs	65	57	32	16	111	34	22	34	31	30
Largest contig (bp)	343,800	689,511	1,524,845	1,557,022	1,557,022	371,701	1,100,025	1,517,151	1,524,846	1,445,065
Total length (bp)	5,712,199	5,635,190	5,849,366	4,967,627	5,280,964	5,280,964	5,569,359	5,726,433	5,840,294	5,711,641
N50 (bp)	188,024	202,973	686,293	1,390,346	1,390,346	79,882	668,640	1,443,580	686,293	690,213
N75 (bp)	101,676	128,558	230,621	407,875	407,875	47,434	362,608	207,366	186,286	206,180
GC (%)	48.88	45.28	45.36	43.65	43.65	53.62	34.96	45.40	45.36	45.38
Genes	5,274	4,975	5,169	5,118	5,255	5,446	5,635	5,075	5,162	5,011
Protein coding	5,159	4,913	5,044	4,882	5,060	5,310	5,459	4,950	5,033	4,907
Non-coding	72	22	75	64	72	75	58	79	79	61
Coverage (×)	1082.86	1082.86	1305.91	1437.35	1471.72	1041.85	1047.66	1269.73	1359.57	1160.26
SRA identifiers	SRX20866903	SRX20866904	SRX20866905	SRX20866906	SRX20866907	SRX20866908	SRX20866909	SRX20866910	SRX20866911	SRX20866912
GenBank identifiers	GCA_030412165.1	GCA_030412275.1	GCA_030412235.1	GCA_030412265.1	GCA_030412255.1	GCA_030412175.1	GCA_030412285.1	GCA_030412135.1	GCA_030412145.1	GCA_030412155.1

## Data Availability

This Whole Genome Shotgun project has been deposited at DDBJ/ENA/GenBank under the BioProject accession number PRJNA988090. The genome sequences are deposited under BioSample accession numbers SAMN35999008 to SAMN35999017 and raw sequences under SAMN36017836 to SAMN36017845. The SRA and GenBank identifiers are described in Table 1. RASTtk annotations are available in zenodo doi: 10.5281/zenodo.8087953.
